# Regional MRI volumetry using NeuroQuant versus visual rating scales in patients with cognitive impairment and dementia

**DOI:** 10.1002/brb3.3397

**Published:** 2024-02-04

**Authors:** Karin Persson, Maria L. Barca, Trine Holt Edwin, Lena Cavallin‐Eklund, Gro Gujord Tangen, Hanneke F. M. Rhodius‐Meester, Geir Selbæk, Anne‐Brita Knapskog, Knut Engedal

**Affiliations:** ^1^ The Norwegian National Centre for Ageing and Health Vestfold Hospital Trust Tønsberg Norway; ^2^ Department of Geriatric Medicine Department of Clinical Neuroscience Oslo University Hospital Oslo Norway; ^3^ Karolinska Institutet Stockholm Sweden; ^4^ Department of Rehabilitation Science and Health Technology, Faculty of Health Science Oslo Metropolitan University Oslo Norway; ^5^ Alzheimer Center Amsterdam, Neurology Vrije Universiteit Amsterdam Amsterdam The Netherlands; ^6^ Amsterdam Neuroscience, Neurodegeneration Amsterdam The Netherlands; ^7^ Department of Internal Medicine, Geriatric Medicine Section Vrije Universiteit Amsterdam, Amsterdam UMC Amsterdam The Netherlands; ^8^ Faculty of Medicine University of Oslo Oslo Norway

**Keywords:** automatic regional volumetry, cognitive impairment, dementia, MRI, NeuroQuant, visual rating scale

## Abstract

**Background and purpose:**

The aims were to compare the novel regional brain volumetric measures derived by the automatic software NeuroQuant (NQ) with clinically used visual rating scales of medial temporal lobe atrophy (MTA), global cortical atrophy‐frontal (GCA‐f), and posterior atrophy (PA) brain regions, assessing their diagnostic validity, and to explore if combining automatic and visual methods would increase diagnostic prediction accuracy.

**Methods:**

Brain magnetic resonance imaging (MRI) examinations from 86 patients with subjective and mild cognitive impairment (i.e., non‐dementia, *n* = 41) and dementia (*n* = 45) from the Memory Clinic at Oslo University Hospital were assessed using NQ volumetry and with visual rating scales. Correlations, receiver operating characteristic analyses calculating area under the curves (AUCs) for diagnostic accuracy, and logistic regression analyses were performed.

**Results:**

The correlations between NQ volumetrics and visual ratings of corresponding regions were generally high between NQ hippocampi/temporal volumes and MTA (*r* = −0.72/−0.65) and between NQ frontal volume and GCA‐f (*r* = −0.62) but lower between NQ parietal/occipital volumes and PA (*r* = −0.49/−0.37).

AUCs of each region, separating non‐dementia from dementia, were generally comparable between the two methods, except that NQ hippocampi volume did substantially better than visual MTA (AUC = 0.80 vs. 0.69). Combining both MRI methods increased only the explained variance of the diagnostic prediction substantially regarding the posterior brain region.

**Conclusions:**

The findings of this study encourage the use of regional automatic volumetry in locations lacking neuroradiologists with experience in the rating of atrophy typical of neurodegenerative diseases, and in primary care settings.

## Abstract 2

The aims of this study were compare the novel regional brain volumetric measures derived by the automatic software NeuroQuant® (NQ) with clinically used visual rating scales of medial temporal (MTA), frontal (GCA‐f), and posterior (PA) brain regions. The NQ volumetrics correlated well with the visual rating scales, had similar or even better diagnostic discriminatory power, and in posterior regions it added precision to the diagnostic prediction.

## 1 INTRODUCTION

The need for efficient and cost‐effective diagnostic tools to assist in the clinical evaluation of cognitive decline and its underlying etiologies is urgent due to the rising prevalence of dementia and the potential availability of new treatments for Alzheimer's disease (AD) in the near future (Perneczky et al., 2023; World Health Organization, 2022). Structural magnetic resonance imaging (MRI) of the brain has gained increased relevance during the last years and is now not only used to exclude non‐dementia etiologies but also to support in finding evidence for disease‐specific atrophy patterns. While visual rating scales to evaluate regional structural changes of the most relevant brain regions have been in use for the last decades (Koedam et al., 2011; Pasquier et al., 1996; Scheltens et al., 1992), the scales are subjective and rather coarse. Visual ratings of the frontal (global cortical atrophy‐frontal scale, GCA‐f) and posterior (posterior atrophy scale, PA) brain regions are known to be challenging with lower intra‐ and interrater reliability compared to ratings of medial temporal lobe atrophy (MTA), and with less diagnostic value of the PA for the oldest patients (Ferreira et al., 2015; Koedam et al., 2011; Rhodius‐Meester et al., 2017). In addition, as many as one‐third of radiologists have been found to lack complete confidence in the visual rating methods (Vernooij et al., 2019). Support of automatic quantification methods might aid in this challenge. Thus, automatic and data‐driven methods have been developed and are widely used in research but to a lesser extent incorporated into everyday clinical routine (Pemberton et al., 2021). The adoption of commercially available clinically feasible automatic methods in clinical practice might be hampered by their insufficient clinical validation. Indeed, a 2021 review found that some methods had been validated against manual segmentation and visual rating scales, but none of the included methods presented evidence as to how the tools should be integrated into clinical settings or how they should contribute to the diagnostic workup (Pemberton et al., 2021).

At the Memory Clinic at Oslo University Hospital (OUH), Norway, the automatic volumetric method NeuroQuant (NQ) (Brewer et al., 2009) has been in use since 2009. During the first years of use, only a limited number of brain regions were assessed by the method. In previous studies, the hippocampus volume as reported by NQ and visual ratings with the MTA scale have been found to correlate well and have equivalent discriminatory power in separating AD from non‐dementia patients (Min et al., 2017; Persson et al., 2018). Currently, newer versions of the NQ software produce volumetric measures of more than 50 brain regions, including the clinically relevant frontal, parietal, occipital, and temporal regions (NeuroQuant, n.d.). To the best of our knowledge, no previous NQ study has validated these novel regional measures.

The main aim of the present study was to compare the novel regional volumetric measures of NQ with the well‐known and clinically validated visual rating scales through correlation analyses and to compare their diagnostic accuracy and explore if combining both methods would add value to diagnostic prediction.

## 2 METHODS

### 2.1 Participants

Participants were recruited from the Memory Clinic at OUH and had been enrolled in The Norwegian Registry of Persons Assessed for Cognitive Symptoms (NorCog) with written consent for their data in the registry to be used for research (Medbøen et al., 2022). All patients received standardized workup and were referred to brain MRI according to clinical indication. For the present study, we included patients who had been visually rated by an experienced neuroradiologist (Lena Cavallin‐Eklund) as part of a previous study (Persson et al., 2017). Of these 218 patients, 88 had been scanned at the research MRI scanner at OUH, where the NQ software was installed. Selection for referral to this specific research scanner, and not to another MRI scanner, was done at random, mainly based on availability and capacity. Of the 88 patients, two were categorized as healthy and were excluded from the sample.

### 2.2 Diagnoses

The 88 patients were diagnosed according to clinical diagnostic criteria by two physicians (Karin Persson and Trine Holt Edwin) retrospectively based on all clinical information from NorCog and the medical records from the clinical examination (Medbøen et al., 2022). The Jessen criteria were used to diagnose subjective cognitive decline (SCD, *n* = 21), and NIA/AA criteria were used to diagnose mild cognitive impairment (MCI, *n* = 20) and dementia (*n* = 45) (Albert et al., 2011; Jessen et al., 2014). The majority of the patients with dementia had AD according to the NIA/AA criteria (either probable AD or possible AD mixed with other pathology, *n* = 34) (Albert et al., 2011). To diagnose other dementia etiologies, the following criteria were used: Rascovsky et al. (2011) for frontotemporal dementia (FTD, *n* = 2), McKeith et al. (2017) for dementia with Lewy bodies (DLB, *n* = 3), Emre et al. (2007) for Parkinson disease dementia (PDD, *n* = 3), and VASCOG criteria for vascular dementia (VaD, *n* = 1) (Sachdev et al., 2014). Additionally, two patients had unspecified dementia. Patients with SCD or MCI are hereafter referred to as “non‐dementia”.

### 2.3 MRI acquisition and analysis

All MRIs were performed on the same GE SIGNA HDxt 3T scanner (GE Healthcare). The scans were rated by an experienced neuroradiologist (Lena Cavallin‐Eklund) and analyzed with NQ (version 3, CorTechs Labs/University of California) (Brewer et al., 2009).

Visual ratings of the medial temporal, frontal, and posterior brain lobes were carried out according to three well‐validated scales using T1‐sequences. The Scheltens scale was used to rate the atrophy of the medial temporal lobe (MTA) on a scale from zero to four (Scheltens et al., 1992). The mean score of the left and right side was used in this study. The GCA‐f scale was used to rate the global cortical atrophy of the frontal lobes on a scale from zero to three (Pasquier et al., 1996), and the PA scale was used to rate cortical atrophy of the posterior brain region (mainly parietal lobes and parietooccipital sulcus) on a scale from zero to three (Koedam et al., 2011). On all three scales, a higher score indicates more atrophy.

NQ produces volumetric measures of several brain regions including the hippocampus as well as joint volumetric measures of the temporal, frontal, parietal, and occipital cortical regions. Volumes are presented as both raw volumes and proportions of intracranial volume (ICV), as well as percentiles calculated based on data from healthy individuals (CorTechs Labs., Inc, n.d.). In this study, we report regional volumes as the total volume of both hemispheres as a proportion of ICV. We used hippocampi and temporal volumes as the NQ correlates to MTA, frontal volume as the NQ correlates to GCA‐f, and parietal and occipital volumes as the NQ correlates to PA.

### 2.4 Statistics

Analyses were carried out using IBM SPSS Statistics for Windows (version 27, Armonk) with a significance level set at .05. Independent samples *t*‐test and *χ* ^2^ tests were used for descriptive comparisons. Pearson correlation coefficients were produced to compare the visual and volumetric normal distributed measures, interpreted according to Cohen, that is, *r* < 0.1—very small, 0.1 ≤ *r* < 0.3—small, 0.3 ≤ *r* < 0.5—moderate, and *r* ≥ 0.5—large (Cohen, 1988). Receiver operating characteristics (ROC) analyses, calculating the area under the curve (AUC), were performed to validate the performance of each MRI classifier (i.e., the three visual measures and the five NQ volumetrics) in distinguishing dementia from non‐dementia (both SCD and MCI) and from SCD alone. An AUC is generally regarded as poor if 0.5 < AUC < 0.7, acceptable if 0.7 ≤ AUC < 0.8, excellent if 0.8 ≤ AUC < 0.9, and outstanding if ≥0.9 (Hosmer et al., 2013). To investigate the potential synergistic value of combining both visual and NQ volumetric methods in enhancing diagnostic prediction, we conducted three logistic regression analyses. These three analyses used dementia/non‐dementia as the outcome variable, with each analysis focusing on one specific brain region. Model a included visual measures, model b included automatic measures, and model c included both visual and automatic measures. The risk of multicollinearity increases when correlated variables with *r* above 0.5 or 0.8 are included in the same model (Shrestha, 2020), and the correlation coefficients between MRI measures of each region varied between −0.37 and −0.72 (Table 2). However, as the main purpose of including correlated variables in the same model was to compare the explained variance of each model using Nagelkerke *R* ^2^, the risk of multicollinearity, especially relevant for the analysis of the temporal measures (*r* = −0.72), was taken into consideration but regarded acceptable. All regression models were adjusted for age and sex.

### 2.5 Ethics

All patients gave written informed consent to be included in NorCog including that all information collected at their examinations at the clinic, including supplemental data such as MRI results, can be used for research. The present study was approved by the Regional Committee of Medical Research Ethics of the South‐East Norway Regional Health Authority (REC South‐East number 2019/79).

## 3 RESULTS

Patient characteristics are presented in Table 1. The mean (SD) age was 71.9 (8.0) in the dementia group and 67.9 (10.1) in the non‐dementia group (*p* = .046), and 22 (49%) versus 14 (34%) were females (*p* = .166). Patients with dementia had significantly more atrophy on all MRI measures compared to the non‐dementia patients (*p* ≤ .003).

**TABLE 1:** Patient characteristics.

	SCD (*n* = 21)	MCI (*n* = 20)	Dementia (*n* = 45)
Age (years)	65.2 (9.7)	70.8 (9.9)	71.9 (8.0)
Females, (*n*, %)	7 (33%)	7 (35%)	22 (49%)
Education (years)	14.5 (3.5)	14.2 (3.2)	12.9 (3.6)
MMSE (score)	29.3 (0.7)	26.7 (2.9)	22.3 (5.8)
CDR‐SB (score)	0.3 (0.4)	1.7 (1.0)	5.8 (3.2)
MTA mean (score)	1.1 (0.6)	1.5 (0.9)	2.0 (1.0)
GCA‐f (score)	0.3 (0.5)	0.6 (0.8)	0.8 (0.6)
PA (score)	0.3 (0.6)	0.4 (0.1)	1.0 (0.8)
Hippocampi/ICV (%)	0.51 (0.07)	0.42 (0.09)	0.40 (0.08)
Temporal/ICV (%)	8.5 (0.7)	8.1 (0.9)	7.3 (1.0)
Frontal/ICV (%)	11.5 (0.9)	11.0 (1.4)	10.5 (0.9)
Parietal/ICV (%)	7.1 (0.6)	6.9 (0.8)	6.4 (0.8)
Occipital/ICV (%)	3.9 (0.3)	3.8 (0.4)	3.5 (0.4)

*Note*: All continuous variables are expressed as mean (SD).

Abbreviations: CDR‐SB, clinical dementia rating scale sum of boxes; GCA‐f, global cortical atrophy‐frontal; ICV, intracranial volume; MCI, mild cognitive impairment; MMSE, mini mental status examination; MTA, medial temporal lobe atrophy; PA, posterior atrophy; SCD, subjective cognitive decline.

Correlations between all MRI measures are presented in Table 2. The highest correlation was found between the NQ volumetry of the hippocampi and the visual rating of MTA (*r* = −0.72). For all regional NQ volumetrics, the correlation was highest with the visual rating scale of the corresponding region, that is, between NQ hippocampi and temporal volumes and the MTA (*r* = −0.72/−0.65), between NQ frontal volume and the GCA‐f (*r* = −0.62), and between NQ parietal and occipital volumes and the PA (*r* = −0.49/−0.37).

**TABLE 2:** Correlations between NQ volumetric measures and visual rating measures.

	Visual rating measures		
NQ volumetric measures	MTA mean	GCA‐f	PA
Hippocampi/ICV	**−0.72**	−0.49	−0.34
Temporal/ICV	**−0.65**	−0.47	−0.39
Frontal/ICV	−0.60	**−0.62**	−0.44
Parietal/ICV	−0.38	−0.41	**−0.49**
Occipital/ICV	−0.32	−0.21*	**−0.37**

*Note*: Bold values indicate a correlation between measures from equivalent regions of the two measures. Pearson *r*, *p* < .01 for all except *p .059.

Abbreviations: GCA‐f, global cortical atrophy‐frontal; ICV, intracranial volume; MTA, medial temporal lobe atrophy; PA, posterior atrophy.

To validate the performance of the different MRI classifiers, ROC analyses were performed using dementia versus non‐dementia as the outcome (Table 3, column a). The AUCs of the NQ hippocampi and temporal volumes were 0.74 and 0.80 (“acceptable/excellent”), respectively, and the AUC of MTA was 0.69 (“poor”); the AUC of NQ frontal volume was 0.70 (“acceptable”), and the AUC of GCA‐f was 0.69 (“poor”); finally, the AUCs of NQ parietal and occipital volumes were 0.74 and 0.73, respectively, and the AUC of PA was 0.72 (“acceptable”). When using dementia versus SCD as the outcome, all AUCs were within the “acceptable” range, except that NQ hippocampi and temporal volumes increased to “excellent” (0.86 and 0.85) (Table 3, column b). Conjoining all five NQ volumes gave an AUC of 0.77 for dementia versus non‐dementia and 0.84 for dementia versus SCD.

**TABLE 3:** Discriminatory properties. (a) Dementia versus non‐dementia and (b) dementia versus subjective cognitive decline (SCD).

	(a)		(b)	
	AUC (95% CI)	*p*	AUC (95% CI)	*p*
Visual measures				
MTA mean	0.69 (0.58−0.81)	.002	0.74 (0.62−0.85)	.002
GCA‐f	0.69 (0.58−0.81)	.002	0.74 (0.61−0.87)	.002
PA	0.72 (0.61−0.83)	<.001	0.74 (0.61−0.87)	.002
NQ volumetrics				
Hippocampi/ICV	0.74 (0.63−0 85)	<.001	0.86 (0.77−0.95)	<.001
Temporal/ICV	0.80 (0.71−0.89)	<.001	0.85 (0.76−0.94)	<.001
Frontal/ICV	0.70 (0.60−0.81)	.002	0.77 (0.65−0.89)	<.001
Parietal/ICV	0.74 (0.63−0.84)	<.001	0.78 (0.67−0.90)	<.001
Occipital/ICV	0.73 (0.63−0.84)	<.001	0.79 (0.68−0.90)	<.001

ROC analyses.

Abbreviations: AUC, area under the curve; CI, confidence interval; GCA‐f, global cortical atrophy‐frontal; ICV, intracranial volume; MTA, medial temporal lobe atrophy; NQ, NeuroQuant; PA, posterior atrophy.

In line with the ROC results, the explained variances were higher in the logistic regression model b (NQ volumetric measures) than in model a (visual measures) for all three brain regions (Tables 4, 5, 6). The Nagelkerke R2 of model c (including both NQ volumetric and visual measures) reached a higher level than that of models a and b only regarding the posterior brain region .

**TABLE 4:** Associations with dementia/non‐dementia. Models a, b, and c include temporal measures.

	Unadjusted		Adjusted model a		Adjusted model b		Adjusted model c	
	OR (95% CI)	*p*	OR (95% CI)	*p*	OR (95% CI)	*p*	OR (95% CI)	*p*
MTA mean	2.4 (1.4−4.1)	**.002**	2.4 (1.2−4.5)	**.010**			1.3 (0.6−2.8)	.580
Hippocampi/ICV	0.6 × 10^−4^ (1.9 × 10^−7^–1.7 × 10^−2^)	**<.001**			0.001 (3.4 × 10^−8^–17.7)	.162	0.003 (4.8 × 10^−8^–157.1)	.291
Temporal/ICV	0.3 (0.2−0.5)	**<.001**			0.3 (0.1−0.6)	**.002**	0.3 (0.1−0.6)	**.003**
Nagelkerke *R* ^2^				.21		.40		.40

*Note*: Logistic regression analyses. Models a, b, and c are adjusted for age and sex.

Abbreviations: CI, confidence interval; ICV, intracranial volume; MTA, medial temporal lobe atrophy; OR, odds ratio.

**TABLE 5:** Associations with dementia/non‐dementia. Models a, b, and c include frontal measures.

	Unadjusted		Adjusted model a		Adjusted model b		Adjusted model c	
	OR (95% CI)	*p*	OR (95% CI)	*p*	OR (95% CI)	*p*	OR (95% CI)	*p*
GCA‐f	3.0 (1.4−6.5)	**.005**	2.7 (1.1−6.3)	**.025**			1.8 (0.7−4.8)	.214
Frontal/ICV	0.5 (0.3−0.8)	**.003**			0.5 (0.3−0.8)	**.010**	0.5 (0.3−1.0)	.060
Nagelkerke R^2^				.17		.20		.22

*Note*: Logistic regression analyses. Models a, b, and c are adjusted for age and sex.

Abbreviations: CI, confidence interval; GCA‐f, global cortical atrophy‐frontal; ICV, intracranial volume; OR, odds ratio.

**TABLE 6:** Associations with dementia/non‐dementia. Models a, b, and c include posterior measures.

	Unadjusted		Adjusted model a		Adjusted model b		Adjusted model c	
	OR (95% CI)	*p*	OR (95% CI)	*p*	OR (95% CI)	*p*	OR (95% CI)	*p*
PA	3.2 (1.6−6.3)	**<.001**	3.2 (1.6−6.4)	**.001**			2.2 (1.0−4.8)	**.038**
Parietal/ICV	0.3 (0.2−0.6)	**<.001**			0.4 (0.1−0.9)	**.026**	0.5 (0.2−1.2)	.123
Occipital/ICV	0.1 (0.02−0.3)	**<.001**			0.2 (0.04−1.1)	.063	0.2 (0.04−1.2)	.086
Nagelkerke *R* ^2^				.27		.34		.39

*Note*: Logistic regression analyses. Models a, b, and c are adjusted for age and sex.

Abbreviations: CI, confidence interval; ICV, intracranial volume; OR, odds ratio; PA, posterior atrophy.

## 4 DISCUSSION

The correlations between regional automatic NQ volumetric measures and corresponding visual rating scale measures were generally high, with the highest correlation found between the temporal NQ volumes and MTA, and the lowest between the posterior NQ volumes and PA. The discriminatory power of each regional NQ volumetric measure and its corresponding visual rating measure were comparable, except that the NQ volumetry of the hippocampi and temporal regions was substantially better at discriminating dementia from non‐dementia compared to MTA. Combining NQ volumetrics with visual rating measures increased the diagnostic prediction accuracy in posterior brain regions.

In line with previous studies, the highest correlation was found between measures of the medial temporal region. Previous studies have found automatic quantification methods of the medial temporal region to correlate well with both manual and visual measurements (Koikkalainen et al., 2019; Mårtensson et al., 2020), while correlations for frontal regions have been found to be substantially lower (Koikkalainen et al., 2019; Mårtensson et al., 2019). In a study by Koikkalainen et al. (2019), the medial temporal region correlation coefficients were 0.83 and 0.78, substantially higher than ours, and the coefficient for the global cortical atrophy was 0.64, similar to our NQ frontal volume/GCA‐f results. They did not study posterior regions exclusively. To our knowledge, no study using a clinically feasible automatic software has presented data on the parietal or posterior brain regions. The automatic visual ratings of the atrophy model, which is not yet clinically available, present results for the parietal region that are in line with those of the medial temporal region (Mårtensson et al., 2019), that is, higher than the ones we found for the posterior region.

While few previous results using clinically feasible software are available, our current results showing that NQ volumetrics correlate best with their corresponding regional visual rating scale indicate good validity of the NQ volumetric method. Indeed, for all regional NQ volumetrics, the correlation was the highest with the corresponding visual measure. The relatively lower correlation between the posterior measurements could be related to the fact that this region is the most difficult to rate visually (Rhodius‐Meester et al., 2017). However, it could also be potentially caused by less variation in the volumetric and visual rating scale scores of posterior regions related to the majority of the included dementia patients suffering from AD, typically involving mainly the medial temporal region, especially in the early phases of the disease. On the contrary, our patients were young, many suffering from young onset AD where posterior cortical atrophy is often present (Koedam et al., 2010).

All AUCs were comparable or better using NQ volumetry versus visual ratings, indicative of a better ability of NQ to discriminate dementia from non‐dementia. In a previous study including a subsample of the present cohort, we found NQ volumetry of the hippocampus to be better at discriminating AD dementia from non‐dementia (Persson et al., 2018), but no previous study has assessed the validity of the frontal and posterior volumetrics of NQ. In the study by Koikkalainen et al. (2016), another automatic MRI quantification algorithm combining several automatic quantification methods was notably better (higher diagnostic accuracy for AD, FTD, VaD, DLB, and HC) than visual MRI ratings alone.

While NQ performed comparable or better than visual assessments, combining both methods only seemed to affect the prediction accuracy of the posterior brain region. In a study on the effect of adding an automatic volumetry report (Geodesic Information Flows) to a regular radiological evaluation, the diagnostic accuracy (of separating healthy controls, AD and FTD) improved when radiologists were presented with the results of the volume report. However, that study did not analyze the use of the QReport alone (Pemberton et al., 2021). In another study, on separating AD from FTD (*n* = 42), presenting only automatic reports to the radiologist decreased the diagnostic accuracy compared to using visual measures alone, while combining the two increased the accuracy (Vernooij et al., 2018). Overall, it seems the regional NQ volumetrics performed quite comparable to other automatic measures, perhaps somewhat lower in temporal regions, but studies were not comparable in the sample size. While the correlation was lower between posterior measures, and the atrophy of this region being difficult to rate, it was of special interest to find that combining posterior volumetrics to the visual measurement added precision to the prediction of the diagnostic stage.

In the near future, in parallel with, and as a consequence of, the development of the disease‐modifying AD treatment, the need of biomarkers specific to the AD pathological processes will presumably change the role of MRI from being a supporting diagnostic tool to being a tool to aid in diagnostic stratification, prognostic prediction, and therapeutic effect evaluation. Simultaneously, the number of patients needing assessments will increase dramatically (World Health Organization, 2022). Thus, the integration of time‐efficient automated tools with fewer challenges concerning the need of neuroradiological expertise and lower interrater reliability (Mårtensson et al., 2020) will be prompted. We believe the present findings support an increased use of automatic software, but further validations in larger and longitudinally followed‐up cohorts are necessary.

The main limitation of this pilot study is the sample size. However, to the best of our knowledge, it is the first study comparing the clinically important regional volumetrics of NQ against visual rating measures. Patients were diagnosed retrospectively and without the inclusion of biological biomarkers (i.e., beta‐amyloid or phosphorylated tau markers), but this was not regarded as a major drawback for the purpose of validating the method.

To conclude, we found the novel regional NQ volumetrics to correlate well with validated visual rating scales, to have a similar or even better diagnostic discriminatory power, and to add precision to the diagnostic prediction (posterior volumetric measures). We believe these results should be replicated in a larger sample, to ultimately conclude regarding a beneficial use of automatic volumetry in clinical practice.

## AUTHOR CONTRIBUTIONS

**Karin Persson**: Conceptualization; data curation; formal analysis; writing—original draft; writing—review and editing. **Maria Barca**: Data curation; writing—review and editing. **Trine Holt Edwin**: Data curation; writing—original draft; writing—review and editing. **Lena Cavallin‐Eklund**: Data curation; investigation; writing—original draft; writing—review and editing. **Gro Gujord Tangen**: Methodology; writing—original draft; writing—review and editing. **Hanneke F.M. Rhodius‐Meester**: Methodology; writing—original draft; writing—review and editing. **Geir Selbæk**: Project administration; resources; writing—original draft; writing—review and editing. **Anne‐Brita Knapskog**: Investigation; methodology; writing—original draft; writing—review and editing. **Knut Engedal**: Investigation; writing—original draft; writing—review and editing.

## CONFLICT OF INTEREST STATEMENT

Dr. Persson and Dr. Knapskog report work with the Novo Nordisk NN6535‐4730 trial outside the submitted work. Prof. Selbæk reports participation in Roche and Biogen advisory boards. Dr. Rhodius‐Meester performs contract research for Combinostics, and all funding is paid to her institution. The other authors declare no conflicts of interest.

### PEER REVIEW

The peer review history for this article is available at https://publons.com/publon/10.1002/brb3.3397.

## Data Availability

The data that support the findings of this study are available on request from the corresponding author. The data are not publicly available due to privacy or ethical restrictions.
